# High-Dose vs Standard-Dose Amoxicillin Plus Clavulanate for Adults With Acute Sinusitis: A Randomized Clinical Trial

**DOI:** 10.1001/jamanetworkopen.2021.2713

**Published:** 2021-03-23

**Authors:** Jennifer Gregory, Bichtram Huynh, Brittany Tayler, Chaitali Korgaonkar-Cherala, Gina Garrison, Ashar Ata, Paul Sorum

**Affiliations:** 1 Medicine and Pediatrics, Albany Medical Center Hospital, Cohoes, New York; 2 Englewood Health Medical Center, Englewood, New Jersey; 3 Albany Medical College, Albany, New York; 4 Department of Obstetrics-Gynecology, Stony Brook University Hospital, Stony Brook, New York; 5 Department of Pharmacy Practice, Albany College of Pharmacy and Health Sciences, Albany, New York; 6 Department of Surgery, Albany Medical College, Albany, New York; 7 Department of Emergency Medicine, Albany Medical College, Albany, New York; 8 Department of Internal Medicine, Albany Medical College, Albany, New York; 9 Department of Pediatrics, Albany Medical College, Albany, New York

## Abstract

**Importance:**

Acute bacterial sinusitis is common, but currently recommended antibiotic treatment provides minimal benefit.

**Objective:**

To confirm the previous finding that high-dose amoxicillin plus clavulanate (with double the amount of amoxicillin) may be superior to standard-dose amoxicillin plus clavulanate in adults.

**Design, Setting, and Participants:**

This double-blind, comparative-effectiveness randomized clinical trial was conducted from February 26, 2018, through May 10, 2020, at the academic primary care internal medicine and pediatrics practice of Albany Medical Center, located in Cohoes, New York. Participants included adults aged 18 years or older who were prescribed amoxicillin plus clavulanate for acute bacterial sinusitis diagnosed in accordance with the Infectious Diseases Society of America guidelines.

**Interventions:**

Amoxicillin 875 mg with clavulanate 125 mg plus either placebo (standard dose) or amoxicillin 875 mg (high dose) twice a day for 7 days.

**Main Outcomes and Measures:**

The primary efficacy outcome was a global rating of “a lot better” or “no symptoms” at the end of 3 days of treatment using a Global Rating of Improvement scale, with outcomes ranging from 1 (a lot worse) to 6 (no symptoms). The primary adverse effect outcome was severe diarrhea at 3 or 10 days after the start of treatment.

**Results:**

At an unplanned interim analysis prompted by COVID-19 restrictions, 157 of a projected 240 participants had been enrolled (mean age, 48.5 [range, 18.7-84.0] years; 117 women [74.5%]), with 79 randomized to the standard dose and 78 to the high dose; 9 and 12, respectively, withdrew or were lost to follow-up before the assessment of the primary outcome. At day 3, 31 of 70 participants (44.3%) in the standard-dose group reported a global rating of “a lot better” or “no symptoms,” as did 24 of 66 (36.4%) in the high-dose group, for a difference of −7.9% (95% CI, −24.4% to 8.5%; *P* = .35). The study was, therefore, stopped for futility. Diarrhea was common in both groups by day 3, with any diarrhea reported in 29 of 71 participants (40.8%) receiving the standard dose and 28 of 65 (43.1%) receiving the high dose and severe diarrhea reported in 5 of 71 (7.0%) and 5 of 65 (7.7%), respectively.

**Conclusions and Relevance:**

The results of this randomized clinical trial suggest that adults treated for clinically diagnosed acute sinusitis did not appear to benefit from taking high-dose compared with standard-dose amoxicillin plus clavulanate.

**Trial Registration:**

ClinicalTrials.gov Identifier: NCT03431337

## Key Points

**Question:**

Will additional amoxicillin benefit adults with acute bacterial sinusitis treated with standard-dose amoxicillin and clavulanate?

**Findings:**

This randomized clinical trial of 157 patients with sinusitis was stopped early for futility when an interim analysis showed that a global rating of “a lot better” or “no symptoms” at the end of 3 days was reported by 31 of 70 patients (44.3%) who received a standard dose of amoxicillin plus clavulanate but only 24 of 66 patients (36.4%) receiving a high dose.

**Meaning:**

Additional amoxicillin does not appear to benefit adults receiving standard-dose amoxicillin and clavulanate for acute sinusitis.

## Teaser

This randomized clinical trial evaluates the effects of high-dose amoxicillin plus clavulanate vs standard-dose amoxicillin plus clavulanate in adults with acute bacterial sinusitis.

## Introduction

Acute sinusitis is a common outpatient diagnosis and is typically treated with antibiotics even though the current recommended regimen (875 mg of amoxicillin with 125 mg of clavulanate by mouth twice a day)^1^ has been shown in clinical trials to provide only minimal benefit beyond placebo.^2^ The Infectious Disease Society of America (IDSA) recommends using high-dose amoxicillin with clavulanate (containing amoxicillin 2000 mg rather than 875 mg twice a day) only when the prevalence in the community of penicillin-resistant pneumococci exceeds 10%.^1^ However, few primary care clinicians will know this prevalence, and evidence from studies in children of the penetration of amoxicillin into middle ear fluid^3^ and of the treatment of acute sinusitis with high-dose amoxicillin with clavulanate^4^ suggests that higher-dose amoxicillin might provide more benefit to adults as well, even in areas with a low prevalence of penicillin-resistant pneumococci.

A double-blind, placebo-controlled trial was performed from 2014 to 2017 comparing high-dose and standard-dose amoxicillin with clavulanate for adults with clinically diagnosed acute bacterial sinusitis.^5^ That study confirmed that very few individuals carried resistant bacteria. The primary outcome was the participants’ subjective rating of improvement after 3 days of treatment, knowing that most people get better by 2 weeks but value rapid relief and that the benefit of other common treatments—eg, for streptococcal pharyngitis^6^ and influenza^7^—is largely a quicker improvement. The study drug was the extended-release formulation of high-dose amoxicillin with clavulanate. Midway through the trial, the manufacturer stopped producing all formulations of the study drug. The study drug was transitioned to a combination of standard immediate-release amoxicillin with clavulanate (875/125 mg) and immediate-release amoxicillin (875 mg) twice a day. Unexpectedly, the extended-release formulation provided no significant benefit, but the immediate-release formulation did; in the second, immediate-release part of the study, 52% of participants receiving the high dose rated symptoms as “a lot better” at the end of 3 days of treatment vs 34% receiving the standard dose (*P* = .04).

This finding needed, however, to be replicated because (1) it was, arguably, the result of a subgroup analysis that was not initially planned; (2) the number of participants in the immediate-release part of the study was less than half of the whole cohort, even if enough for unadjusted statistical significance; (3) the secondary outcome—the change in the rating of 16 symptoms (the validated Sinonasal Outcome Test-16 [SNOT-16]^8^) from baseline to the end of 3 and 10 days after enrollment—did not show significant improvement; (4) the biological explanation—that the high concentration achieved for a short time by the immediate-release formulation might be needed for sufficient penetration of amoxicillin into sinus fluid—was only hypothetical; and (5) the percentage of patients reporting severe diarrhea at the end of day 3 was unexpectedly^9^ higher for the immediate-release, high-dose group. The current study aimed to confirm the benefits of high-dose, immediate-release amoxicillin with clavulanate and to explore the balance for patients between more rapid clinical improvement vs more common and severe diarrhea.

## Methods

We conducted this randomized, double-blind, comparative-effectiveness, pragmatic clinical trial from February 26, 2018, through May 10, 2020, at the academic primary care internal medicine and pediatrics practice of Albany Medical College in Cohoes, New York. It was approved by the college’s institutional review board, registered at ClinicalTrials.gov prior to starting the recruitment of participants, and funded by the corresponding author. Written informed consent was obtained from each participant. This study adhered to the Consolidated Standards of Reporting Trials (CONSORT) reporting guideline, and the full trial protocol is available in Supplement 1.

### Participants

Patients 18 years or older who presented to the office with sinus symptoms and whom the clinicians chose to treat with antibiotics were eligible if, in the judgment of the treating clinician, they fit one of the IDSA diagnostic categories for acute bacterial sinusitis and if they had no exclusion criterion. The 3 IDSA categories are (1) persistent symptoms of rhinitis, purulent secretions, and/or pain in the face or teeth without improvement (lasting for ≥10 days), (2) severe symptoms or signs of fever of at least 102 °F and nasal discharge or facial pain (lasting for ≥3-4 days), or (3) worsening symptoms or signs characterized by a new onset of fever, headache, or increase in nasal discharge following a typical viral upper respiratory illness that lasted 5 to 6 days and was initially improving (double sickening).

Patients were excluded if they (1) were allergic or intolerant to any penicillin or to amoxicillin and clavulanate; (2) had experienced a serious hypersensitivity reaction to any beta-lactam; (3) had an elevated risk of carrying amoxicillin-resistant bacteria because they had received amoxicillin, penicillin, or another beta-lactam within the past month or were known to have had methicillin-resistant *Staphylococcus aureus*; (4) had chronic or recurrent sinus problems, defined as persistent symptoms of sinus congestion not attributed to nasal allergies, for 8 weeks or more^10^ or 2 or more episodes of antibiotic-treated sinusitis in the past 3 months; (5) were judged to need high-dose amoxicillin and clavulanate or levofloxacin or be sent to the emergency department because of signs of severe infection or compromised immunity; (6) were cognitively impaired and, therefore, unable to give reliable symptom ratings; (7) were pregnant or nursing; (8) had conditions with warnings about amoxicillin, namely, current mononucleosis, chronic kidney disease with a glomerular filtration rate lower than 30, hepatic impairment, history of antibiotic-associated colitis, or use of allopurinol; or (9) had been previously enrolled in the current study.

### Procedures

Our pharmacist (G.G.) prepared the study medications based on randomized dose allocations using Excel (Microsoft Corporation), with prescription bottles labeled only by study number. Enrolling clinicians, study personnel, and patients were blinded to the allotted dosage. The 2 treatment groups, allocated 1:1, were (1) standard dose: 875 mg of amoxicillin with 125 mg of clavulanate plus placebo tablet (containing lactase) twice a day for 7 days and (2) high dose: 875 mg of amoxicillin with 125 mg of clavulanate plus 875 mg of amoxicillin twice a day for 7 days.

At the time of enrollment, participants filled out SNOT-16,^8^ rating 16 symptoms on a scale of 0 (no problem) to 3 (severe problem): need to blow nose, sneezing, runny nose, cough, postnasal discharge, thick nasal discharge, ear fullness, headache, facial pain or pressure, waking up at night, lack of a good night’s sleep, waking up tired, fatigue, reduced productivity, reduced concentration, and frustrated, restless, or irritable. They also indicated their tendencies to get constipation, diarrhea, or vaginal yeast infections (women). They were advised to use acetaminophen and nasal saline for symptomatic relief and were given a list of the questions they would be asked at the end of day 3.

At the end of day 3, the participants provided a Global Rating of Improvement (GRI) on the scale that Garbutt and colleagues^11^ had used as a secondary outcome and to validate the SNOT-16,^8^ with 1 indicating a lot worse; 2, a little worse; 3, the same; 4, a little better; 5, a lot better; and 6, no symptoms. Participants also replied again to the SNOT-16; rated the adverse effects of diarrhea, abdominal pain, vaginal itching and discharge, or other on a scale of 0 indicating none to 3 indicating severe; and indicated whether they had used nasal saline, nasal steroids (known to improve symptoms^12^), or other medications for their sinus symptoms.

At the end of day 10, participants again provided a GRI, responded to the SNOT-16 questions, and reported the severity of any adverse effects. They rated the balance of good effects and bad effects and indicated whether they would take the antibiotic again if needed.

### Analyses

#### Outcomes

The primary efficacy outcome was the percentage of participants in each group assigning a GRI of 5 or 6 (“a lot better” or “no symptoms”) at the end of 3 days of treatment. The secondary efficacy outcomes were the percentage in each group assigning a GRI of 5 or 6 at day 10 and the mean extent of improvement of the total SNOT-16 score at days 3 and 10. The total SNOT-16 score was the sum of all 16 symptoms rated on a scale of 0 (no problem) to 3 (severe problem), with a possible range of 0 to 48. The primary adverse effect outcome was a score of 3 (severe) for diarrhea at day 3 or 10.

#### Sample Size

The previous study^5^ found that 34% of the participants in the standard-dose group reported a GRI of 5 or 6 at the end of 3 days. We estimated that a sample size of 115 in each group would give us 80% power to detect an increase of 18 percentage points in the GRI among those in the high-dose group (ie, an increase to 52%, as reported previously^5^) with an α of .05 for a 2-sided test. We aimed, therefore, to enroll 240 patients (anticipating 10 dropouts before day 3).

#### Statistical Analysis

We compared baseline characteristics and primary and secondary outcomes using χ^2^ tests, Fisher exact tests, and *t* tests as appropriate for differences in proportions and means. We reported 95% CIs and *P* values for the differences in proportions between the 2 groups, and we reported standard deviations (SDs) for the means for the groups as well as 95% CIs and *P* values for the differences between these means. We used the statistical software Stata, version 15.0 (StataCorp LLC) for analysis and assessed statistical significance at the .05 level.

To look for potential confounding factors, we analyzed the effect on the primary outcome of any significant differences between groups in baseline demographic and clinical characteristics. In our unplanned interim analysis, we did a best-case analysis for participants lost to follow-up, classifying those missing in the high-dose arm as a GRI 5 or 6 and in the standard-dose arm as a GRI less than 5. After the decision to stop the trial, we investigated futility further by calculating, under different assumptions, the conditional power of finding the proposed 18% increase in positive outcomes in the high-dose group if we would continue to the planned enrollment of 115 in each group.

We had planned to do repeat analyses by combining the participants in this study and those in the second time period of the initial study (because the methodology was the same). Given the negative results of the current study, however, we looked only at the primary efficacy outcome.

## Results

### Patient Enrollment

Enrollment was slower than expected for 3 reasons: (1) the diversion of many patients to urgent care providers; (2) the need to warn patients of the risk of severe diarrhea found in the previous study; and (3) the onset of COVID-19, which led to many virtual sick visits and fewer outpatient visits overall. In addition, we had an unexpectedly high rate of failure to obtain primary outcome information (21 of 157 enrolled [13.4%]) as a result of 7 participants who dropped out before day 3, 13 who could not be contacted, and 1 who neglected to provide a global rating. Before continuing the study, our statistician (A.A.) performed an unplanned interim analysis to check for futility and found that the primary outcome in the high-dose arm was inferior to that in the standard-dose arm and that it would be futile to expect to find either a clinically or a statistically significant benefit. As a result, we stopped the trial in May 2020.

**Figure 1.**
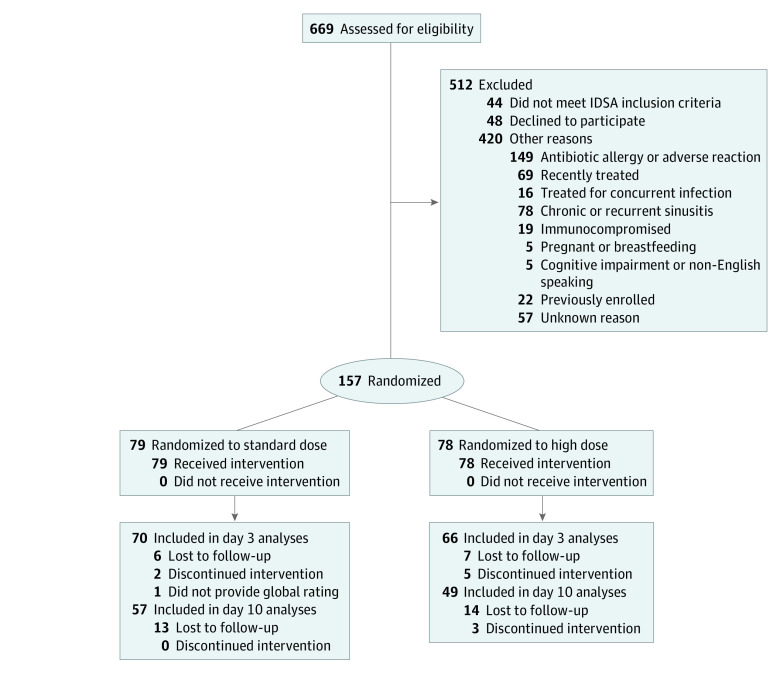
CONSORT Flow Diagram: Patients Treated for Acute Sinusitis IDSA indicates Infectious Disease Society of America.

The CONSORT flow diagram in Figure 1 shows patient enrollment and progress through the study. Of 669 patients 18 years or older treated with antibiotics for acute sinusitis, we enrolled 157 and excluded 512. The reasons for exclusion are listed in Figure 1.

Of the 157 patients enrolled, the mean age was 48.5 (range, 18.7-84.0) years, and 117 (74.5%) were women. Seventy-nine patients were randomized to the standard-dose arm (60 women [76.0%]; mean [SD] age, 48.5 [16.2] years) and 78 to the high-dose arm (57 women [73.1%]; mean [SD] age, 48.5 [15.8] years). The other baseline characteristics are shown in Table 1. The only significant differences between treatment groups were a longer mean [SD] duration of illness in those randomized to the standard dose (17.1 [2.7] days) than the high dose (13.9 [1.4] days) and a higher percentage with allergic rhinitis in the standard-dose group (21 [26.6%] vs 10 [12.8%]). In line with the progressive loss to follow-up and the incomplete collection of data, the results in Table 2 provide the actual numerator and denominator for each outcome.

**Table 1.** Baseline Characteristics of Participants (by Dosage)

Characteristic	No. (%)	
	Standard dose (n = 79)	High dose (n = 78)
Age, mean (SD), y	48.5 (16.2)	48.5 (15.8)
Sex		
Male	19 (24.1)	21 (26.9)
Female	60 (76.0)	57 (73.1)
Comorbidities		
Smoking	7 (8.9)	12 (15.4)
Asthma or COPD	7 (8.9)	13 (16.7)
Diabetes	6 (7.6)	4 (5.1)
Allergic rhinitis	21 (26.6)	10 (12.8)
Heart disease	3 (3.8)	3 (3.9)
History		
Sinusitis	22 (27.9)	19 (24.4)
Hospitalized in past month	0	0
Antibiotics in past month^a^	3 (3.8)	1 (1.3)
Using nasal steroids	19 (24.1)	18 (23.1)
Enrolled in previous study	7 (8.9)	10 (12.8)
Clinical features		
Days of illness, mean (SD)	17.1 (2.7)	13.9 (1.4)
Sinusitis category^b^		
Duration >10 d	58 (76.3)	54 (70.1)
Severe symptoms	6 (7.9)	5 (6.5)
Double sickening	12 (15.8)	18 (23.4)
SNOT-16 total score, mean (SD)^c^	29.4 (8.0)	29.4 (7.4)
Susceptibilities		
Tend to be constipated	19 (25.0)	15 (20.0)
Prone to diarrhea	16 (21.1)	11 (14.7)
Tend to get vaginal yeast infections	15 (25.9)	15 (26.3)

Abbreviations: COPD, chronic obstructive pulmonary disease; SNOT-16, Sinonasal Outcome Test-16.

^a^ Includes antibiotics other than amoxicillin, penicillin, or another beta-lactam.

^b^ The diagnostic category was unclear for 3 patients receiving the standard dose and 1 patient receiving the high dose.

^c^ SNOT-16 total score based on ratings of 16 symptoms on scale of 0 to 3 (total, 0 to 48).

**Table 2.** Outcomes by Dosage

Outcome	Ratio, No./total No. (%)		Difference (95% CI)	*P* value
	Standard dose	High dose		
**Efficacy**				
Global Rating of Improvement score of 5 or 6^a^				
Day 3	31/70 (44.3)	24/66 (36.4)	−7.9% (−24.4% to 8.5%)	.35
Day 10	47/57 (82.5)	35/49 (71.4)	−11.0% (−27.1% to 5.0%)	.18
Decrease in total SNOT-16 score, mean (SD)^b^				
Day 3	12.3 (11.5)	13.3 (8.8)	1.0 (−2.5 to 4.5)	.57
No.	71	65	NA	NA
Day 10	21.4 (10.2)	18.4 (11.4)	−3.1 (−7.2 to 1.1)	.15
No.	56	49	NA	NA
**Adverse effects**				
Any diarrhea				
Day 3	29/71 (40.8)	28/65 (43.1)	2.2% (−14.4% to 18.8%)	.79
Day 10	14/58 (24.1)	14/49 (28.5)	4.4% (−12.3% to 21.2%)	.60
Severe diarrhea				
Day 3	5/71 (7.0)	5/65 (7.7)	0.7% (−8.1% to 9.4%)	>.99
Day 10	2/58 (3.5)	3/49 (6.1)	2.7% (−5.5% to 10.9%)	.66
Any vaginal itching or discharge				
Day 3	11/55 (20.0)	6/45 (13.3)	−6.7% (−21.2% to 7.8%)	.38
Day 10	10/47 (21.3)	9/35 (25.7)	4.4% (−14.2% to 23.1%)	.64
Severe vaginal itching or discharge				
Day 3	0/55 (0)	2/45 (4.4)	4.4% (−1.6% to 10.5%)	.20
Day 10	0/47 (0)	1/35 (2.9)	2.9% (−2.7% to 8.4%)	.43
Overall assessment				
Balance of effects, mean (SD)^c^	2.2 (1.5)	1.8 (1.8)	−0.4 (−1.0 to 0.2)	.22
Take antibiotic again? yes	47/58 (81.0)	34/48 (70.8)	−10.2% (−26.5% to 6.1%)	.22

Abbreviations: NA, not applicable; SNOT-16, Sinonasal Outcome Test-16.

^a^ Assessment of global improvement: 1 = a lot worse, 2 = a little worse, 3 = the same, 4 = a little better, 5 = a lot better, and 6 = no symptoms.

^b^ The SNOT-16 total score is based on ratings of 16 symptoms on a scale of 0 to 3 (total possible score of 0 to 48). Two participants did not fill out the SNOT-16 at baseline.

^c^ Rating at day 10 of the balance of bad vs good effects of antibiotics on a scale from −3 to +3.

### Outcomes by Dosage

#### Primary Efficacy Outcome

**Figure 2.**
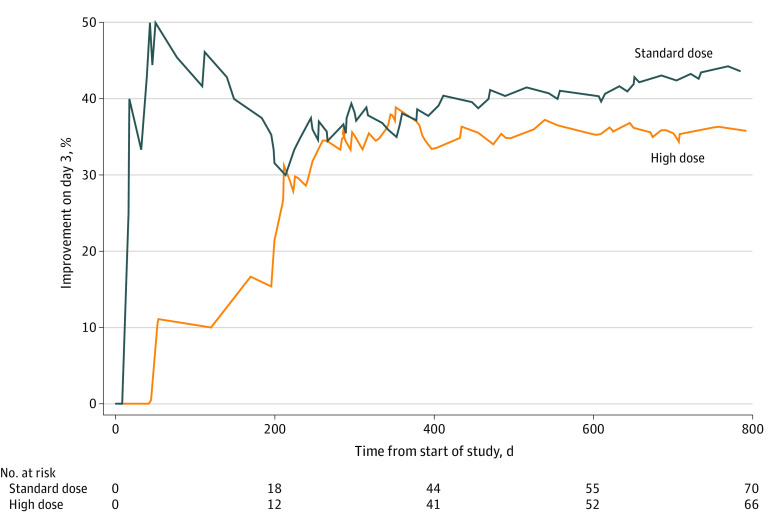
Timeline of Percentages of Primary Efficacy Outcomes in Each Group

The primary efficacy outcome—a global rating of “a lot better” or “no symptoms” (GRI of 5 or 6) after 3 days of treatment—was reached by 31 of 70 patients (44.3%) receiving the standard dose but only 24 of 66 (36.4%) of those receiving the high dose, a statistically nonsignificant difference of −7.9% (95% CI, −24.4% to 8.5%; *P* = .35). Figure 2 shows the timeline of primary outcomes in each group; by midway through the trial, the difference in percentages had become quite consistent.

No significant confounding factors were identified despite differences in baseline characteristics. First, the longer baseline duration of illness for those given the standard dose vs the high dose (mean [SD] of 17.1 [2.7] vs 13.9 [1.4] days; *P* = .03) raised the possibility that more of the patients receiving the standard dose were already on the verge of recovery. However, duration of illness was not associated with a positive efficacy outcome: those with a GRI of 5 or 6 at the end of day 3 had a mean [SD] duration of illness of 15.8 [10.1] days vs 14.8 [8.4] days for the others (*P* = .56). Second, the higher percentage of allergic rhinitis in the standard-dose group (21 [26.6%] vs 10 [12.8%], *P* = .04) raised the possibility of confounding symptoms. Yet even though a slightly lower percentage of those with allergic rhinitis reported a positive primary outcome at day 3 (10 [35.7%] vs 45 [41.7%]), the difference was not significant (*P* = .57). Third, a difference in participants with the diagnostic category of double sickening (12 [15.8%] with the standard dose and 18 [23.4%] with the high dose) had no effect: 11 [40.7%] in the double sickening category and 43 [41.1%] in the other categories had a positive outcome at the end of day 3 (*P* = .98).

In addition, as shown in the eTable in Supplement 2, the dropout rate during the study did not significantly alter the balance in important baseline characteristics of those participants available at the end of days 3 and 10, including age; sex; underlying conditions of smoking, asthma or chronic obstructive pulmonary disease, or allergic rhinitis; nasal steroid use; days of illness; IDSA category; or SNOT-16 score. Furthermore, for those lost to follow-up, even if we assigned negative outcomes to everyone receiving the standard dose and positive outcomes to everyone receiving the high dose, the difference in the primary outcome was not significant: 39.2% (31 of 79) had a negative outcome in the standard-dose group vs 46.2% (36 of 78) in the high-dose group, a difference of 6.9% (95% CI, −8.5% to 22.4%; *P* = .38). The combined primary efficacy outcomes of this study and the corresponding segment of the original study were 39.6% (53 of 134) rating “a lot better” or “no symptoms” at 3 days in the standard-dose groups and 44.2% (57 of 129) in the high-dose groups, for a difference of 4.6% (95% CI, −7.3% to 16.5%; *P* = .45).

#### Secondary Efficacy Outcomes

The secondary efficacy outcomes also showed no significant benefit from high-dose amoxicillin and clavulanate (Table 2). At day 10, 47 of 57 (82.5%) in the standard-dose group and 35 of 49 (71.4%) in the high-dose group had a GRI score of 5 or 6, for a difference of −11.0% (95% CI, −27.1% to 5.0%). The mean (SD) decrease in total SNOT-16 score at day 3 was 12.3 (11.5) for the standard-dose group and 13.3 (8.8) for the high-dose group, for a difference of 1.0 (95% CI, −2.5 to 4.5) and at day 10 was 21.4 (10.2) and 18.4 (11.4), respectively, for a difference of −3.1 (95% CI, −7.2 to 1.1).

A post hoc calculation of the percentage in each group whose change in mean rating in all SNOT-16 symptoms exceeded the minimal clinically important difference of 0.6 (on a scale of 0 to 3) determined by Garbutt and colleagues^8^ found that a mean decrease of 0.6 or more per symptom was reported by 59.2% (42 of 71) of patients in the standard-dose group and 63.1% (41 of 65) of patients in the high-dose group at the end of day 3 (a difference of 3.9%; 95% CI, −12.4% to 20.3%; *P* = .64) and by 89.3% (50 of 56) of standard-dose and 73.5% (36 of 49) of high-dose participants (a difference of −15.8%; 95% CI, −30.6% to −1.0%; *P* = .04) at the end of day 10.

#### Adverse Effects

Adverse effects, particularly diarrhea, were common in both treatment groups (Table 2). At the end of day 3, 57 of 136 participants (41.9%) reported at least some diarrhea, including 29 of 71 (40.8%) receiving the standard dose and 28 of 65 (43.1%) receiving the high dose (a difference of 2.2% [95% CI, −14.4% to 18.8%]), and 10 of 136 participants (7.4%) reported severe diarrhea, including 5 of 71 (7.0%) standard-dose and 5 of 65 (7.7%) high-dose participants (a difference of 0.7% [95% CI, −8.1% to 9.4%]). Reports of some diarrhea decreased at day 10 to 28 of 107 (26.2%), including 14 of 58 patients (24.1%) receiving the standard dose and 14 of 49 patients (28.5%) receiving the high dose (a difference of 4.4% [95% CI, −12.3% to 21.2%]), and of severe diarrhea to 5 of 107 (4.7%), including 2 of 58 (3.4%) and 3 of 49 (6.1%), respectively (a difference of 2.7% [95% CI, −5.5% to 10.9%]).

Among female participants, any vaginal itching or discharge was reported at day 3 by 11 of 55 (20.0%) receiving the standard dose and 6 of 45 (13.3%) receiving the high dose (a difference of −6.7% [95% CI, −21.2% to 7.8%]) and at day 10 by 10 of 47 (21.3%) and 9 of 35 (25.7%), respectively (a difference of 4.4% [95% CI, −14.2% to 23.1%]). Severe vaginal itching or discharge was reported by no standard-dose participants and 3 high-dose participants (2 of 45 [4.4%] at day 3 and 1 of 35 [2.9%] at day 10).

Participants’ indication that they were prone to diarrhea or to vaginal yeast infections was, as shown in Table 3, associated with suffering at least mild symptoms. At day 3, any diarrhea was reported by 16 of 26 participants (61.5%) prone to diarrhea and 41 of 105 participants (39.1%) not prone to diarrhea (*P* = .04). At day 10, any vaginal symptoms were reported by 10 of 24 participants (41.7%) prone to vaginal symptoms and 9 of 53 participants (17.0%) not prone to vaginal symptoms (*P* = .02).

**Table 3.** Effect of Proneness to Symptoms

Symptom	Participants, No./total No. (%)		*P* value
	Prone	Not prone	
Baseline	27	120	NA
Diarrhea			
Any day 3	16/26 (61.5)	41/105 (39.1)	.04
Any day 10	6/21 (28.6)	21/80 (26.3)	.83
Severe day 3	4/26 (15.4)	6/105 (5.7)	.09
Severe day 10	1/21 (4.8)	4/80 (5.0)	.96
Vaginal symptoms			
Any day 3	6/24 (25.0)	11/72 (15.3)	.28
Any day 10	10/24 (41.7)	9/53 (17.0)	.02
Severe day 3	0/24 (0)	2/72 (2.8)	.41
Severe day 10	0/24 (0)	1/53 (1.9)	.50

Abbreviation: NA, not applicable.

#### Overall Assessment and Futility Analysis

Overall, patients who took the standard dose reported a more positive balance between the good and bad effects of the antibiotics—with a mean (SD) rating of 2.2 (1.5) points vs 1.8 (1.8) points on a scale of −3 to +3 (a difference of −0.4 [95% CI, −1.0 to 0.2] points; *P* = .22)—and were more willing to take the antibiotic again (47 of 58 [81.0%] vs 34 of 48 [70.8%], a difference of −10.2% [95% CI, −26.5% to 6.1%]; *P* = .22).

Our post hoc futility analysis indicated that, if we continued to the planned enrollment of 115 in each group, the conditional power of finding the proposed increase of 18% in positive outcomes in the high-dose group would be only 12.9% if the current differences persisted (shown in Figure 2) and still only 45.1% if we assumed that the subsequent outcomes would favor high dose by the hypothesized 18%.

## Discussion

In this randomized clinical trial, we did not find a benefit to patients of treating clinically diagnosed acute bacterial sinusitis with high-dose rather than standard-dose amoxicillin with clavulanate. This finding is disappointing because the IDSA-recommended standard dose^1^ has a limited effect (vs placebo).^2^ This outcome is also surprising in light of the promising results of the previous trial.^5^

Why would we get such very different results? First, the current trial suffered from an incomplete collection of data (although mostly for the secondary outcomes). Yet these missing data cannot explain such a pronounced lack of benefit. A best-case analysis of the missing primary outcomes made only a modest difference. We found no important changes in baseline characteristics in the participants who remained at the end of days 3 and 10, and it seemed futile to continue recruitment to achieve the planned number of participants. Second, the 2 groups were not completely similar at baseline. Standard-dose participants reported a longer average duration of illness; therefore, more of them might have had spontaneous improvement unrelated to antibiotic dose by the end of day 3. However, the actual impact of duration of illness appeared to be minimal. In addition, even if an effect of allergic rhinitis in diminishing the response to antibiotics had been found, it would have reduced primary outcomes in more participants in the standard-dose group. Third, one or both of the studies may have suffered from a randomly skewed, nonrepresentative sample of participants. Whatever the explanation, the addition of the current participants to those from our previous study reduced the benefit of high-dose amoxicillin with clavulanate to statistical nonsignificance.

Three secondary findings are of interest. First, the frequency of diarrhea and vaginal yeast infections was quite high, even though most cases were mild.^9^ Second, patients who reported they were prone to adverse effects were more likely to experience them. Whether or not this outcome represented a self-fulfilling prophecy, this information would seem important when discussing with patients the pros and cons of antibiotic treatment. Third, the large number of patients with presumed penicillin allergy (Figure 1) underlines the need for more widespread testing for penicillin allergy. As shown in multiple studies,^13,14^ most of these patients do not have, or no longer have, an allergy to penicillin and can benefit from having the option of treatment with a penicillin.

### Limitations

Our study has several limitations. First, as discussed above, we stopped the study early after enrolling only 157 (65%) of a planned 240 participants, and we failed to obtain follow-up data on the primary outcome from 21 participants (13.4%) and on secondary outcomes from a greater number. Nonetheless, our best-case analyses showed that the finding of no benefit from high-dose amoxicillin with clavulanate was resilient, and our post hoc futility analyses confirmed this outcome. Second, differences between the 2 groups in baseline characteristics—initially only the number of days of illness and the percentage reporting allergic rhinitis—could have increased as patients were lost to follow-up. For the primary outcome, however, neither duration of illness nor underlying allergic rhinitis had significant effects, and we found no important changes in the baseline characteristics of those who remained in the study at the end of days 3 and 10. Third, the use of a subjective rating as the primary outcome allowed distortion by unrelated personal factors. Nonetheless, subjective outcomes are not only important to patients but, as indicated in the Introduction, are commonly used in studies of treatment of infectious diseases,^6,7^ including acute sinusitis.^8,11^ We chose not to obtain data on more objective outcomes, such as days out of work or school, judging that such data would be underpowered, but future studies could look at these outcomes.

## Conclusions

How can clinicians help their patients with presumed acute bacterial sinusitis as defined by the current IDSA guidelines? This randomized clinical trial found that adding more amoxicillin to the standard, but minimally beneficial, treatment with amoxicillin and clavulanate does not appear to be the answer. Quinolones have shown some efficacy^15^ but are not without risks, including black box warnings.^16^ The best policy may be, therefore, to emphasize the use, also in accordance with IDSA guidelines,^1^ of saline flushes and nasal steroids.^12^

## Notes

Supplement 1. **Trial Protocol**

Supplement 2. **eTable.** Changes in Baseline Characteristics in Each Group by Days 3 and 10

Supplement 3. **Data Sharing Statement**
