# Supplementary material for: High-Dose vs Standard-Dose Amoxicillin Plus Clavulanate for Adults With Acute Sinusitis: A Randomized Clinical Trial
Source: JAMA Netw Open. 2021 Mar 23;4(3):e212713. doi: 10.1001/jamanetworkopen.2021.2713 (PMC7988367; doi:10.1001/jamanetworkopen.2021.2713)
Supplement: Supplement 1. — Trial Protocol [file jamanetwopen-e212713-s001.pdf]

Research Protocol

Treatment of Acute Sinusitis with High-Dose vs. Standard-Dose Amoxicillin/Clavulanate: A Confirmation Study

Investigators

Principal Investigator: Paul Sorum, MD, PhD
Co-Investigators: Danielle Wales, MD, MPH; Jennifer Gregory, MD; Bichtram Huynh, MD; Gina Garrison, PharmD

Background and Purpose

Acute sinusitis is a common out-patient diagnosis and is commonly treated with antibiotics even though the current recommended regimen, amoxicillin/clavulanate 875/125,[1] has been shown in clinical trials to provide only minimal benefit beyond placebo.[2] Evidence from studies in children of the penetration of amoxicillin into middle ear fluid[3] and of the treatment of acute sinusitis[4] suggested that a higher dose of amoxicillin would provide more benefit to adults without increasing side effects. We performed in 2014-17, therefore, a double-blind placebo-controlled trial of high-dose vs. standard-dose amoxicillin/clavulanate for clinically diagnosed acute bacterial sinusitis.[5] The study drug was the extended-release formulation of amoxicillin/clavulanate. Midway through the trial, the manufacturer stopped producing both the brand-name and the generic forms of the study drug. We were forced, therefore, to use instead for the high-dose arm a combination of standard immediate-release amoxicillin/clavulanate plus immediate-release amoxicillin. To our surprise, we found that the extended-release formulation provided no significant benefit, but the immediate-release formulation did; with 52% rating their symptoms as "a lot better" at the end of 3 days of treatment (the primary endpoint) vs. 34% ($p = 0.04$).

This finding needs, however, to be replicated because 1) it was, arguably, the result of an analysis not initially planned and of a sub-group; 2) the number of participants in the immediate-release part of the study was not large (less than half of the whole cohort), even if enough for statistical significance; 3) the secondary outcome, the change in the rating of 16 symptoms (the validated SNOT-16) from baseline to the end of 3 and 10 days of treatment, did not show a significant improvement; 4) the biological explanation of the difference in outcomes (that the high concentration achieved, if only for a short time, by the immediate release might be needed for penetration of amoxicillin into sinus fluid) was only hypothetical; and 5) the percent of patients reporting "severe" diarrhea at day 3 was higher for the immediate-release high-dose group than for the others (which was also a surprise since other studies suggested that diarrhea is largely caused by the clavulanate, which was the same for all groups). The balance for patients between more rapid clinical improvement versus more common and severe adverse effects needs to be explored further. One problem is that patients with adverse effects from high-dose amoxicillin may not be able to appreciate that their sinus symptoms improved faster than if they had taken standard-dose.

The Infectious Disease Society of America (IDSA) recommends using high-dose amoxicillin/clavulanate when the prevalence in the community of penicillin-resistant pneumococci is >10%.[1] Few primary care clinicians will know this prevalence. By performing anterior nasal cultures on the first two-thirds of our participants in the initial

trial, we confirmed our expectation that very few of our patients are colonized with penicillin-resistant pneumococci. We will not need to repeat this part of the study.

If we confirm that supplementing standard, immediate-release amoxicillin-clavulanate 875/125 bid with standard, immediate-release amoxicillin 875 bid results in as substantial a benefit as in the initial study, we will be able to provide patients with a treatment that is clearly more efficacious than placebo and that is also quite inexpensive and easily available. If we find that the increase in adverse effects caused by the supplemental amoxicillin does not outweigh, in patients' judgments, the added benefits of treatment, we may change the way acute bacterial sinusitis is treated.

Study Design and Methods

Randomized, double-blind, comparative-effectiveness trial

Site: the Albany Medical College Internal Medicine and Pediatrics primary care practice in Latham, NY

Duration of study: 2 ½ years (November 2017-April 2020)

Investigators

Faculty: Paul Sorum, MD, PhD; Danielle Wales, MD, MPH; Gina Garrison, PharmD

Medicine-Pediatrics residents: Jennifer Gregory, MD; Bichtram Huynh, MD

Albany Medical College student volunteers: Jennifer Cha; Chaitali Korgaonkar; Laura Stanfel

Participants

Adults 18 years and older who are patients at the Albany Medical Center Internal Medicine and Pediatrics Practice in Latham, NY.

Inclusion criteria

1. Clinical diagnosis of acute bacterial sinusitis in accordance with the guidelines of the Infectious Disease Society of America, i.e., fitting into one of 3 categories:
    a. Persistent symptoms of rhinitis, purulent secretions, and/or pain in face or teeth and not improving (lasting for $\geq$ 10 days); or
    b. Severe symptoms or signs of fever $\geq$ 102 degrees F and nasal discharge or facial pain (lasting for $\geq$ 3-4 days); or
    c. Worsening symptoms or signs characterized by a new onset of fever, headache, or increase in nasal discharge following a typical viral URI that lasted 5-6 days and was initially improving ("double-sickening").
2. Patients who participated in the initial study will be eligible. Having chronic or recurrent sinus infections was, and will again be, an exclusion criterion, but it would decrease external validity to exclude patients who occasionally get sinusitis and might, therefore, have participated in the past 3 years. We are able to obtain different looking antibiotic and placebo pills, so that we can include even patients who knew or who think they knew which formulation they took in the first study. We will, however, plan a secondary analysis of those who were and were not in the initial study.

Exclusion criteria

1. Patients who were enrolled previously in the current study

2. Allergy or intolerance to any penicillin (oral penicillin, amoxicillin, or dicloxacillin or IV penicillin, ampicillin, oxacillin, nafcillin, carbenicillin, ticarcillin, or piperacillin) or to amoxicillin/clavulanate
3. Serious hypersensitivity reaction (e.g., anaphylaxis or Stevens-Johnson syndrome) to any beta lactam
4. Elevated risk of amoxicillin-resistant bacteria
    a. Amoxicillin, penicillin, or other beta-lactam within the past month (not, as in the IDSA guidelines [2012], any antibiotics)
    b. Known to have had MRSA
5. Chronic or recurrent “sinus” problems. Defined as persistent symptoms of “sinus” congestion, not attributed to nasal allergies, for 8 weeks or more (Mayo Cinic, 2014) or 2 or more episodes of antibiotic-treated “sinusitis” in past 3 months. These patients are at risk of anatomical or immunological abnormalities and of harboring antibiotic resistant organisms.
6. Need to use high-dose amoxicillin/clavulanate or levofloxacin or to send to ED or to hospitalize because of
    a. Signs of severe infection
    b. Immunocompromise
7. Cognitive impairment, so unable to give reliable symptom ratings (even if a health proxy can give consent)
8. Pregnant women and nursing mothers. Pregnancy is Category B, so we do not need to perform a pregnancy test prior to enrolling pre-menopausal women; but known pregnancy will be an exclusion because amoxicillin/clavulanate has been demonstrated as safe in pregnant animals but not yet in pregnant women, and in practice (as in the previous trial), both clinicians and pregnant women hesitate to participate in experiments unless it is important to include pregnant women as a category. Nursing will be an exclusion because of lack of information even though it is not likely to cause a problem.
9. Drug warnings
    a. Taking allopurinol (which increases substantially the risk of rash)
    b. Current mononucleosis (because of amoxicillin-induced rash)
    c. Chronic kidney disease stage 4 with estimated GFR <30
    d. Hepatic impairment (not including isolated transaminase elevated < 2 times upper limit of normal)
    e. History of antibiotic-associated colitis (C. diffiile)

Time in study

10-11 days (or a few days longer if difficult to make telephone contact at day 10)

Drop-outs

Participants can exit the study at any time: a) by going to Urgent Care or the Emergency Department if very sick (calling our office beforehand if possible, in accordance with office policy); b) by indicating this at the day 3 telephone call; or c) by calling the office or (after hours) the physician on call at any time

If appropriate, the office or on-call physician can switch the antibiotic (in accordance with ISDA guidelines) to doxycycline or levofloxacin

Interventions

Randomization:

Participants will be randomized 1:1 by the pharmacist (GG) and her pharmacy students, who will prepare the study medication in bottles labeled only by study # and who will keep a list of study # and dosage that will be revealed to the investigators only after the completion of the study.

Treatment arms:

1. Standard dose: amoxicillin/clavulanate 875/125 + placebo tablet (OTC lactase) bid x 7 days
2. High dose: amoxicillin/clavulanate 875/125 + amoxicillin 875 bid x 7 days

Measurements:

1. Global rating of improvement: on the scale 1=a lot worse, 2= a little worse, 3=the same, 4=a little better, 5=a lot better, 6=no symptoms (at telephone calls at the end of days 3 and 10)
2. SNOT-16: rating of current condition of 16 symptoms on a scale of 0 = no problem to 4 = severe problem (at enrollment and at telephone calls at days 3 and 10)
3. Additional question at bottom of baseline SNOT-16: do you often have trouble with a) constipation, b) diarrhea, c) (for women) vaginal yeast infections (yes or no)?
4. Rating of adverse effects (diarrhea, abdominal pain, [for women] vaginal itching and discharge, and rash on a scale of 0=none to 3=severe (at days 3 and 10)
5. Were you treated for vaginal yeast infection (if so, when and how, and did it help) and for diarrhea (if so, when and how, and did it help)? (at day 10)
6. How many doses of antibiotic did you not take? If you stopped early, why? (Felt so much better, side effects, forgot, lost medication) (at day 10)
7. How would you balance the good effects and the bad effects of the antibiotic on a scale of -3=bad effects MUCH greater than good effects; -2=bad effects SOMEWHAT greater than good effects; -1= bad effects A LITTLE greater than good effects: 0=bad effects and good effects about equal; +1=good effects A LITTLE greater than bad effects; +2=good effects SOMEWHAT greater than bad effects; +3=good effects MUCH greater than bad effects (at day 10)
8. Would you take this antibiotic again (yes, no, uncertain). Any further explanation of response beyond the previous question? (at day 10)
9. What dosage do you think you took? And why? (looked up or recognized pills, degree of beneficial effect on sinus symptoms, level of severity of side effects) (at day 10)
10. Drop-outs will be asked how many doses they took and why they are dropping out of the study (fear of adverse effects, actual experience of side effects, quick improvement, no improvement)

Endpoints

1. Primary efficacy endpoint: percent giving a global rating of improvement of 5 or 6 after 3 days of treatment
2. Secondary efficacy endpoints:
   a. Percent giving a global rating improvement of 5 or 6 at day 10

   b. Change in SNOT-16 average score from baseline (at enrollment) to 3 and 10 days after enrollment (with minimal clinically important change = 0.5 units of the scale from 0 to 4))
   c. Percent having change in mean SNOT-16 item score at day 3 and 10 $\geq$ .5 (minimal clinically significant difference)
3. Adverse effect endpoints:
   a. Percent having each of adverse effects and having level 3 (severe) of each of side effects 3 and 10 days after enrollment
   b. Percent having side effects in a) of those declaring themselves prone or not to have 1) diarrhea and 2) vaginitis (predictive power)
4. Overall judgments::
   a. Mean rating on balance of good and bad effects at day 10
   b. Distribution of ratings of balance (using different cutoffs)
   c. Distribution of judgments about whether they would take the antibiotic again
5. Impact of using the web: if any significant number of participants use the web to enter their responses, we will compare the answers to the endpoints of web vs. non-web participants
6. Impact of potentially confounding factors:
   a. Use of nasal steroids (which can improve symptoms[6]
   b. Use of other antibiotics in the past month (altering nasal flora)
   c. Discovery of the identity of the pills (unblinding)

Study Materials

1. Posters in waiting and examining room
2. Enrollment form (filled out by enrolling physician)
3. Day 0 SNOT-16 questionnaire (given by nurses to all patients with respiratory symptoms and, if not filled out already, filled out by participants at enrollment)\
4. Consent Form
5. Day 3 and Day 10 telephone or web questions [except for the additional day 10 questions asked over the telephone or on the website] (handed out to participants at enrollment)
6. Directions for nasal saline and contact information (handed out at enrollment)
7. Telephone scripts or web forms at end of days 3 and 10
8. Drop-out form

Study Flow

1. Nurse gives the patient with respiratory symptoms a SNOT-16 form to fill out and mentions the study to the patient.
2. Physician does the standard clinical assessment; decides if the patient is likely to have sinusitis (according to IDSA guidelines) and might benefit from antibiotics; determines if the patient meets inclusion criteria for the study and has no exclusion criterion (as shown on the Enrollment Form); and explains the study to the patient.

3. If the patient will be treated with antibiotics for acute sinusitis but will not be a participant in the trial, the physician will fill out the Treated Outside of Study part of the Enrollment Form and leave it on the designated spot in the Pod.
4. If the patient is willing, the physician gives the Consent Form to the patient, explains what involved, and allows the patient to read it, ask questions, and decide to enter the study or not.
5. If the patient enrolls in the study, the physician, nurse, or pharmacy student
    a. Puts a patient sticker on the next study # in the Participant List in the locked study room;
    b. Obtains the bag of study medications with that study # (prepared in advance by the pharmacist [GG]) and, as required by NYS law, writes on the bottles the patient's name and address, the physician's name, and the date;
    c. Fills out the Enrollment Form: writes the study # on it, on the SNOT-16 form, and on a study folder; puts the forms in the study folder and leaves it in the locked study room; and brings the bag of study medications back to the examining room.
6. The physician then
    a. Gives the participant the study medication, with directions how to take the pills; gives the participant the Day 3 and Day 10 telephone questions with the study # written on them (without the questions added to the Day 10 form) with a request to fill them out after 3 and 10 days just in case the study personnel do not succeed in making contact on time;
    b. Gives the participant the option of filling out the forms online, using AMC's membership in Qualtrics (in which the participant is identified by study number only), and, if desired, has the participant write down his or her email address and tells the participant that we will send by email the directions for the secure website (but also will telephone him or her);
    c. Asks the participant if he or she wants a text message reminder and, if so, has him or her to write down the telephone number for the text (if different from the main number);
    d. Shows the participant the directions for symptomatic treatment.
7. The residents are responsible for making the telephone calls themselves or assigning them to the medical student members of the team. The residents telephone or send AMC emails to the students to provide the names, telephone numbers and dates of required telephone calls.
8. At the ends of day 3 and 10, the participant is called by a member of the study team and asked the questions on the day 3 and day 10 telephone forms. If the participant wants a text reminder and/or wants to fill out the form online, the study team member sends the text and/or email with the link. The study team member enters the data from the web-based form or from the telephone call into the restricted database on the AMC Med-Ped Y drive (with participants identified only by study number) and also fills out the forms to be placed with the other study materials.
9. If the participant sends to us (by e-mail, fax, or mail) a completed day 3 or 10 questionnaire and also provides answers over the telephone and if the written and

verbal answers differ, we will use the written version (unless the participant directs us not to).

10. If the participant asks the study team member for a call from the office, that request is communicated to the on-call physician for the Medicine-Pediatrics practice.
11. If a participant indicates at the day 3 call or at another time that he or she is dropping out of the study, the clinician, medical student, or staff member asks why, fills out the Drop-out form in paper and on the shared electronic database on the Y drive, and (if needed) notifies one of the study team members.
12. After the study has been completed, the data analyzed, and a study report accepted for publication, we will mail to each participant a summary of the study results and an indication of the dosage she or he took. We will then destroy the patient registry with its identifying information.

Data Analyses

Sample size:

To have a power of 80% to find again an increase in the primary endpoint from 34% to 52% with an alpha of 0.05 %, we would need 230 patients (115 in each group). To find an increase only to 50%, we would need 292 patients (146 in each group). Our aim is, therefore, to enroll at least 240 and ideally 300 patients. We enrolled 315 patients in the previous study during an equivalent time period.

Outcomes:

Using SPSS, we will calculate

1. Chi-square analyses of the differences between the two arms in percentages of global ratings, adverse effects, balance between good and bad effects, willingness to take the antibiotic again, use of nasal steroids, and correct guesses of the dose (with alpha set at 0.05).
2. T-test analyses of the differences between the two arms in the changes of average SNOT-16 item rating from baseline to the end of day 3 and to the end of day 10 and in the mean rating of balance between good and bad effects at day 10 (alpha 0.05)
3. Subgroup analyses for 1) and 2) of those who did vs. did not participate in the initial study.
4. Repeat analyses combining a) the participants in this study and b) the participants in Time Period 2 of the initial study.

Human subjects

Risks

1. Adverse physical effects

   In the initial study the primary adverse effects were diarrhea and (in women) vaginal discharge or itching. In the second time period, comparing the antibiotic formulations used in the current study, the increases in these adverse effects associated with a doubling of the amoxicillin were:

   a. Diarrhea
      i. At day 3; any diarrhea 47.37 % vs. 30.65% (p=0.06); severe diarrhea 15.79% vs. 4.84% (p=0.05)

        ii. At day 10: any diarrhea 17.32% vs. 12.00% (p=0.45); severe diarrhea 3.85% vs. 0 (p=0.16)
    b. Vaginal itching or discharge
        i. At day 3: any symptoms 15.91% vs. 7.84% (p=0.22); severe symptoms 2.27% vs. 1.96% (p=0.92)
        ii. At day 10: any symptoms 23.68% vs 17.50% (p=0.50); severe symptoms 0 vs. 2.56% (p=0.33)
2. Flaws in decision making
    a. Under-treatment: the risk of enrolling in the study a very sick patient who should be either treated with levofloxacin or sent to the ED. To guard against this, the enrolling physician must attest that the patient is not too sick to receive out-patient amoxicillin/clavulanate.
    b. Over-treatment: the risk of enrolling patients who ought not to receive antibiotics. To minimize this, the enrolling physician must indicate to which of the 3 IDSA categories of acute bacterial sinusitis the patient belongs.
    c. Coercion: the risk that, because we are their primary care office, patients who prefer not to be in a study will allow themselves to be enrolled. In the previous study, however, we found many patients not afraid to say no.
3. Breach of confidentiality

    This is very unlikely to be a problem (see below). Nonetheless, since the study database indicates the presence or absence of the comorbidities of smoking, asthma or COPD, diabetes, and heart disease, it is conceivable, though unlikely, that someone might use this information to target the participant for marketing or other purposes. If a data breach does take place, we will immediately notify the AMC risk management office.

Benefits

1. Patients receiving the extra dose of amoxicillin may benefit from a more rapid rate of improvement.
2. Other patients are likely to benefit if clinicians know whether or not to treat acute sinusitis with high-dose amoxicillin/clavulanate.

Confidentiality

1. The enrolling physician, office nurse, or pharmacy student will place the patient's label next to the study # in the study patient registry. The patient number will be written on a study folder as well as on the enrollment form, the Consent form, and the baseline SNOT-16 that will be placed in that study folder (along with the day 3 and 10 telephone forms, as well as the Drop-out form if needed). The study patient registry and the files will be kept in a locked room.
2. Communication between team members of patient names and telephone numbers will be either verbally or via Albany Med email (hence secure).
3. The participants who chose to enter their answers to the day 3 and 10 questionnaires electronically will receive an email from the study team member with directions on how to access the secure Qualtrex website with their study number and will enter their data on it, identified only by study #. The study team

member will be able to access this data and transfer it to the study-wide database on the Y drive.
4. The study-wide shared electronic database for the entry of day 3 and 10 responses will be kept in the sinusitis study folder on the AMC Med-Ped Y drive, with access limited to members of the study team only, and will identify participants by study # only i.e. will contain no personal identifying information.
5. The material in the participant’s study file will contain no information that is at all likely to be embarrassing or compromising to the patient.
6. The study team members are clinicians (physicians, office nurses, the pharmacy professor (GG), and pharmacy students) who are part of the practice and already have routine access to the patients’ full medical records. The students from the Albany College of Pharmacy and Health Sciences have full access to patient records during their blocks in the office and, under the supervision of the pharmacy professor, regularly review and counsel patients about their medications. The medical students will not have access to the patient registry or the study files, although they will of course know the names of the participants whom they contact.
7. Study data will ultimately be entered into an Excel database. Individual participants will be identified by study #, not by name; data will include sex and age (but not date of birth). The database will be kept in the sinusitis study folder of the AMC Y drive, with access limited to study team members.
8. When the trial is complete, the data analyzed, and the report accepted for publication, we plan to notify participants of the study results and of the dosage they received and then to destroy the patient registry. We will not destroy the files and database, identified only by study #, so that, if needed, we can do further analyses.

Costs

Funds will come from the AMC Med-Ped Research Fund

1. Medications (for 300 participants)
    a. Amoxicillin/clavulanate 875/125: 4200 pills = $840
    b. Amoxicillin 875: 2100 pills = $525
    c. Placebo (lactase): 2100 pills = $300
    d. Medication bottles and caps: 600 = $210
2. Duplication of forms = $350
3. Letters to participants after publication of study results = $300
4. Total: $2,175

References

1. Chow AW, Benninger MS, Brook I, et al. IDSA clinical practice guideline for acute bacterial rhinosinusitis in children and adults. Clin Infect Dis. 2012;54(8):e72-e112.
2. Lemiengre MB, van Driel ML, Merenstein D, Young J, De Sutter AIM. Antibiotics for clinically diagnosed acute rhinosinusitis in adults. Cochrane Database Syst Rev. 2012;10:CD006089.

3. Canafax DM, Yuan Z, Deka K, Russlie HQ, Giebink GS. Amoxicillin middle ear fluid penetration and pharmacokinetics in children with acute otitis media. Pediatr Infect Dis J. 1998;17(2):149-156.
4. Wald ER, Nash D, Eickhoff J. Effectiveness of amoxicillin/clavulanate potassium in the treatment of acute bacterial sinusitis in children. Pediatrics. 2009;124(1):9-15.
5. Matho A, Mulqueen M, Tanino M, et al. High-dose versus standard-dose amoxicillin/clavulanate for clinically-diagnosed acute bacterial sinusitis: a randomized clinical trial. 2017; unpublished manuscript.
6. Zalmanovici Trestioreanu A, Yaphe J. Intranasal steroids for acute sinusits. Cochrane Database of Systematic Reviews 2013, Issue 12, Art No CD005149.
