# Supplementary material for: High-Dose vs Standard-Dose Amoxicillin Plus Clavulanate for Adults With Acute Sinusitis: A Randomized Clinical Trial
Source: JAMA Netw Open. 2021 Mar 23;4(3):e212713. doi: 10.1001/jamanetworkopen.2021.2713 (PMC7988367; doi:10.1001/jamanetworkopen.2021.2713)
Supplement: Supplement 2. — eTable. Changes in Baseline Characteristics in Each Group by Days 3 and 10 [file jamanetwopen-e212713-s002.pdf]

Supplemental Online Content

Gregory J, Huynh B, Tayler B, et al. High-dose vs standard-dose amoxicillin plus clavulanate for adults with acute sinusitis: a randomized clinical trial. *JAMA Netw Open.* 2021;4(3):e212713. doi:10.1001/jamanetworkopen.2021.2713

eTable. Changes in Baseline Characteristics in Each Group by Days 3 and 10

This supplemental material has been provided by the authors to give readers additional information about their work.

© 2021 Gregory J et al. *JAMA Network Open.*

eTable. Changes in Baseline Characteristics in Each Group by Days 3 and 10

Baseline Characteristic	Time Period	Standard Dose		High Dose		P-value
		No. in group	*Mean (95% CI)*	*No. in group*	*Mean (95% CI)*	
Age (years)	Baseline	79	48.5 (44.8–52.1)	78	48.5 (45.0-52.1)	.98
	Day 3	71	49.3 (45.4-53.2)	65	49.1 (45.1-53.1)	.94
	Day 10	56	48.2 (43.7-52.6)	49	50.3 (45.6-55.0)	.51
			Percent (95% CI)			
Female	Baseline	79	76.0% (66.5-85.4)	78	73.1 (63.2-82.9)	.68
	Day 3	71	77.5% (67.8-87.2)	65	69.2 (58.0-80.5)	.28
	Day 10	56	80.4% (70.0-90.8)	49	71.4 (58.8-84.1)	.28
			No. (%)		*No. (%)*	
Smoking	Baseline	79	7 (8.9%)	78	12 (15.4%)	.23
	Day 3	71	7 (9.9%)	65	11 (16.9%)	.14
	Day 10	56	4 (7.1%)	49	6 (12.2%)	.14
Asthma/COPD	Baseline	79	7 (8.9%)	78	13 (16.7%)	.16
	Day 3	71	7 (9.9%)	65	12 (18.5%)	.22
	Day 10	56	4 (7.1%)	49	9 (18.4%)	.14
Allergic rhinitis	Baseline	79	21 (26.6%)	78	10 (12.8%)	.04
	Day 3	71	20 (28.2%)	65	8 (12.3%)	.03
	Day 10	56	16 (28.6%)	49	5 (10.2%)	.03
Nasal steroid use	Baseline	79	19 (24.1%)	78	18 (23.1%)	1.0
	Day 3	71	18 (25.4%)	65	16 (24.6%)	1.0
	Day 10	56	17 (30.4%)	49	11 (22.5%)	.39
			Mean (95% CI)		*Mean (95% CI)*	
Days of illness	Baseline	65	17.1 (14.4-19.8)	70	13.9 (12.5-15.3)	.03
	Day 3	59	16.9 (13.9-19.8)	57	13.7 (12.1-15.2)	.06
	Day 10	46	15.5 (12.6-18.4)	42	13.8 (12.0-15.6)	.33

© 2021 Gregory J et al. *JAMA Network Open.*

SNOT-16 total score	Baseline	78	29.4 (27.6-31.2)	77	29.4 (27.7-31.1)	.99
	Day 3	71	29.6 (27.8-31.5)	65	29.8 (28.1-31.6)	.87
	Day 10	56	30.4 (28.5-32.4)	49	30.5 (28.5-32.5)	.96
**Time Period**	**IDSA category**		*No. (%)*		*No. (%)*	
Baseline	Duration > 10 days	76	58 (76.3%)	77	54 (70.1%)	.49
	Severe symptoms	76	6 (7.9%)	77	5 (6.5%)	
	Double sickening	76	11 (16.2%)	77	18 (23.4 %)	
Day 3	Duration > 10 days	68	53 (77.9%)	64	44 (68.8%)	.48
	Severe symptoms	68	4 (5.9%)	64	4 (6.3%)	
	Double sickening	68	11 (16.2%)	64	16 (25.0%)	
Day 10	Duration > 10 days	53	41 (77.4%)	48	31 (64.6%	.31
	Severe symptoms	53	4 (7.6%)	48	4 (8.3%)	
	Double sickening	53	8 (15.1%)	48	13 (27.1%)	

© 2021 Gregory J et al. *JAMA Network Open.*
