# Supplementary material for: High-Dose vs Standard-Dose Amoxicillin Plus Clavulanate for Adults With Acute Sinusitis: A Randomized Clinical Trial
Source: JAMA Netw Open. 2021 Mar 23;4(3):e212713. doi: 10.1001/jamanetworkopen.2021.2713 (PMC7988367; doi:10.1001/jamanetworkopen.2021.2713)
Supplement: Supplement 3. — Data Sharing Statement [file jamanetwopen-e212713-s003.pdf]

Data Sharing Statement

Gregory. High-Dose vs Standard-Dose Amoxicillin Plus Clavulanate for Adults With Acute Sinusitis. *JAMA Netw Open*. Published March 23, 2021. doi:10.1001/jamanetworkopen.2021.2713

Data
Data available: Yes
Data types: Deidentified participant data
How to access data: sorump@amc.edu
When available: With publication

Supporting Documents
Document types: None

Additional Information
Who can access the data: Researchers who request the data
Types of analyses: For any medically-indicated purpose
Mechanisms of data availability: After I approve the request
